# What is the structure of our infrastructure? A review of UK light microscopy facilities

**DOI:** 10.1111/jmi.13076

**Published:** 2022-01-10

**Authors:** Georgina Fletcher, Kurt I. Anderson

**Affiliations:** ^1^ BioImaging, UK and the Royal Microscopical Society 37/38 St Clements Oxford, OX4, 1AJ; ^2^ The Francis Crick Institute, 1 Midland Rd, London NW1 1AT

**Keywords:** BioImaging UK, career development, core facilities, Euro‐BioImaging, German BioImaging, light microscopy, research funding

## Abstract

Core Facilities and Technology Platforms are increasingly important components of the science research landscape. However, data on facility operations and staff careers are lacking to inform their development. Here we have surveyed 114 people working in 46 light microscopy (LM) facilities within the United Kingdom. Our survey explores issues around career progression, facility operations and funding. The data show that facilities are substantial repositories of equipment and knowledge which adapt to meet the needs of their local environments. Our report highlights the challenges faced by facility managers, institutions and funders in evaluating facility performance and devising strategies to maximise the return on research funding investment.

## 1 INTRODUCTION

Biological science has become increasingly specialised and multi‐disciplinary. High‐impact projects often rely on multiple, expensive, advanced technologies, which are collectively beyond the ability of an individual laboratory to fund and master.^1^ This has driven a trend towards the concentration of equipment and know‐how in technology groups which typically serve a local scientific community such as a university, institute or department. DNA and protein sequencing were two of the earliest such technologies to be provided through core facilities, with electron microscopy facilities following in the late 1980s and light microscopy facilities emerging in the late 1990s. In Europe, the delivery of LM as a research service has been supported through the European Light Microscopy Initiative (ELMI), which held its first session dedicated to core services in 2006 and has organised a full‐day satellite meeting since 2012. The first meeting of UK Light Microscopy Facilities (UKLMF) Managers was a small gathering of managers in York in January of 2006, which has since grown to an annual event of several hundred people, comprising facility managers, staff and commercial partners. The early UKLMF meetings led to the development of the UK LM facilities database to promote contact and awareness among LM facilities in both the life and material sciences. This database was first developed at the University of York and subsequently hosted by the Royal Microscopical Society (RMS) (BioImagingUK, 2015).

The development of LM as a research service has progressed unsystematically. The individual groups who initially set up and ran LM facilities learned their own lessons along the way. Organisations such as the RMS, ELMI, Core Technologies for Life Sciences (CTLS), the Association of Biomolecular Resource Facilities (ABRF), German BioImaging and France BioImaging subsequently served to bring core facility staff together to share experience and develop good practice. Facility staff have also tried to more formally capture and disseminate the lessons they have learned. Early reports focused on operational aspects of how to set up and run LM facilities, including how to lay out and equip a facility; aspects of equipment, user and staff management; safety and consideration of facility funding (Anderson et al., 2007).^1^ Later, as operational data accumulated, the field began to consider funding and performance metrics, which have become important to the facility review process.^2^, ^3^, ^4^ LM core facility staff have also drawn upon their experience working with a wide range of collaborators to publish on training,^5^ the development of rigorous experimental approaches to light microscopy^6^, ^7^, ^8^ and best practice in reporting microscopy methods.^9^ These reports and others emphasise the important role of core facilities in promoting quality control and scientific reproducibility.

BioImaging UK (BIUK) was formed in November 2009 as an attempt by the broader UK imaging community (LM, EM and medical) to organise itself in response to the challenge of Euro‐BioImaging (EuBi). Although the United Kingdom was a founding member of the EuBi ERIC, it has only recently begun to consider how to organise light microscopy as a form of national infrastructure able to interact with EuBi. In order to set up a useful imaging infrastructure, we must understand the state of our existing resources and the imaging needs of the scientific community. As a first step in determining our future direction, this survey is a high‐level attempt to characterise our present state by asking, ‘What is the structure of our infrastructure?’ In addition to typical metrics of facility performance, our survey explores issues around career progression, funding and how facilities are integrated within their institutional environments.

## 2 METHODS

The survey was conducted online using SurveyMonkey between the 7 April 2020 and the 12 May 2020(inclusive), coinciding with the first national lockdown in the United Kingdom resulting from the Coronavirus pandemic. The survey was tested on a group of six London LM facility managers and revised before being sent out to the wider community. The final survey link was distributed over the BIUK Jisc Email List, the RMS Facility Database List and widely posted online via BIUK's Twitter and LinkedIN accounts. All respondents were anonymous and IP addresses were not tracked. The survey contained questions in the following formats: open‐field text, open‐field numbers, checkboxes and multiple‐choice. Survey data were analysed using Python and graphed using Plotly Express. The Survey Monkey report included 170 responses, of which 114 were unique and valid and 113 were complete. Thirteen responses from outside the United Kingdom and 3 responses from UK facilities not managing LM were excluded from the report but serve to indicate the level of interest in this survey. The survey questions were divided into three sections (Supplementary Document). The first section aimed to collect information about facility staff themselves (13 questions). The second section was designed to allow operational comparison among UK facilities and with Figures 2 and 3 of the German BioImaging survey^2^ (17 questions). The third section probed how facilities are structured, funded and reviewed (8 questions). All responders were able to reply to the first section concerning career progression; however only self‐declared facility managers had access to the questions concerning Operations and Facility Organization. Of the 114 unique responses, 58 were from facility staff and 56 were from facility managers. However, 10 facility managers did not progress to the second two parts of the survey providing information about their facilities. Furthermore one facility response was incomplete, so that some questions report on 45 facilities rather than 46. As a matter of data privacy, we are unable to publish the raw data from the survey; however we will gladly work with researchers wanting to ask specific questions of the data.

### 2.1 Career progression

**FIGURE 1:**
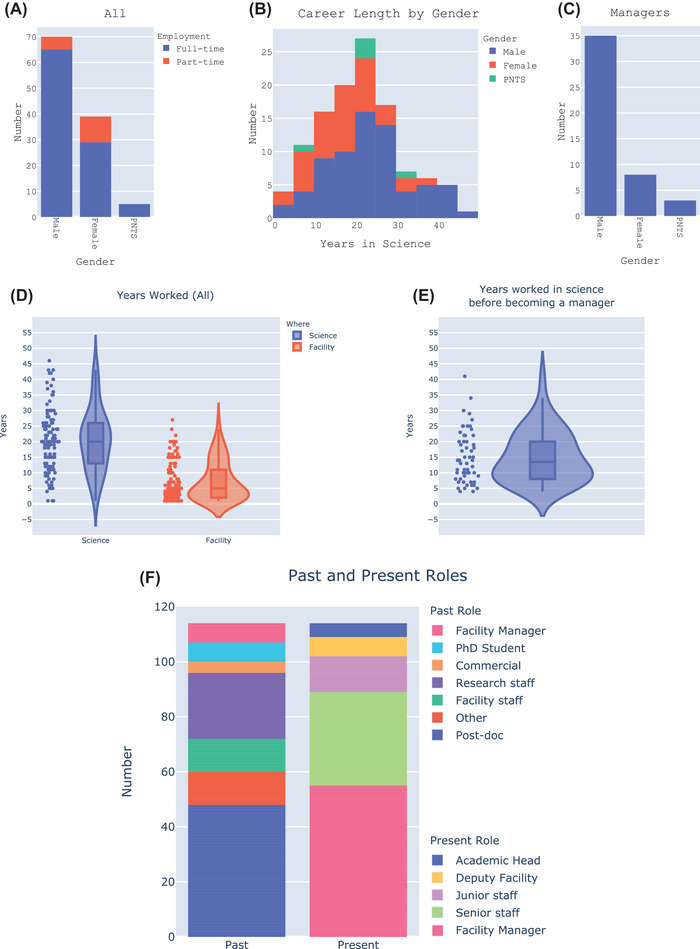
Histograms showing (A) the gender balance of all full and part time employees, (B) career length of staff by gender and (C) the gender balance of facility managers. Unless otherwise noted, ‘staff’ includes managers. (D) Violin plots showing the number of years worked by all staff in science and in light microscopy facilities. (E) Violin plot showing the number of years managers worked in science before becoming a manager. (F) Bar chart showing past and present roles across all survey responses

Of the 114 responses, 70 declared male, 39 declared female and 5 preferred not to say (PNTS), resulting in a male to female gender ratio of just under 2:1 (Figure 1A). The ratio of males to females at different career stages is broadly similar until about 25 years in science, after which there are proportionately fewer females (Figure 1B). The ratio of male to female managers was just over 4:1 (Figure 1C). Eight per cent of all males, 26% of all females and 0% of PNTS were employed part‐time (Figure 1A).

**FIGURE 2:**
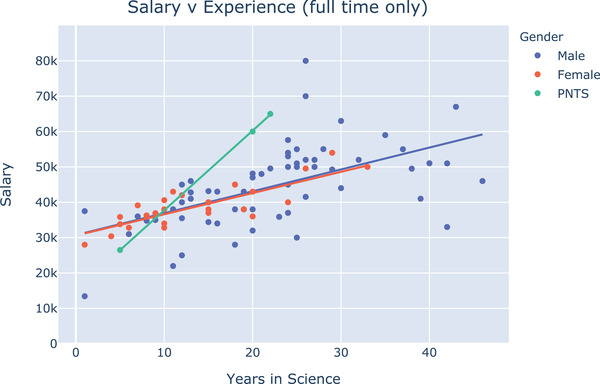
Scatter plot showing salary as a function of years worked in science for male, female and ‘prefer not to say’ (PNTS) staff. Trendlines indicate similar rates of salary progression between males and females (trendline parameters are listed in the Supplementary Table S1)

LM facility staff are highly experienced; 50% of the staff surveyed had between 13 and 26 years of experience working in science, and between 2 and 11 years working in a facility (Figure 1D). Unless otherwise noted ‘staff’ is used here to include managers and is equivalent to FTE. Half of the facility managers surveyed had worked between 8 and 20 years in science before becoming a manager (Figure 1E). The lower proportion of females having over 25 years of experience most likely contributes to the greater gender imbalance among managers. Forty‐one per cent of all staff had worked as a post‐doc in their previous role whereas 24% had worked as research staff (research assistant, technician or officer; Figure 1F). Seventeen per cent previously worked in a facility (as staff or manager) and very few facility staff came directly into their present position from a PhD or commercial role (2.2% each). The majority (80%) are on permanent contracts and most (70%) are on a technical services career track (Supplementary Figure S1A). The five most common job descriptors (and their frequencies) were: manager (39), facility (34), senior (22), imaging (20) and research (17) (Supplementary Figure S1B). When part‐time workers are excluded, the correlation between salary and years of experience was similar for males and females (Figure 2).

### 2.2 Operations

Although 55 responders said they were facility managers, only 46 went on to provide operational information about their facilities (and one of these responses was incomplete). This represents just over half of the 83 biological LM facilities listed on the RMS Facilities database (BioImagingUK, 2015).

**FIGURE 3:**
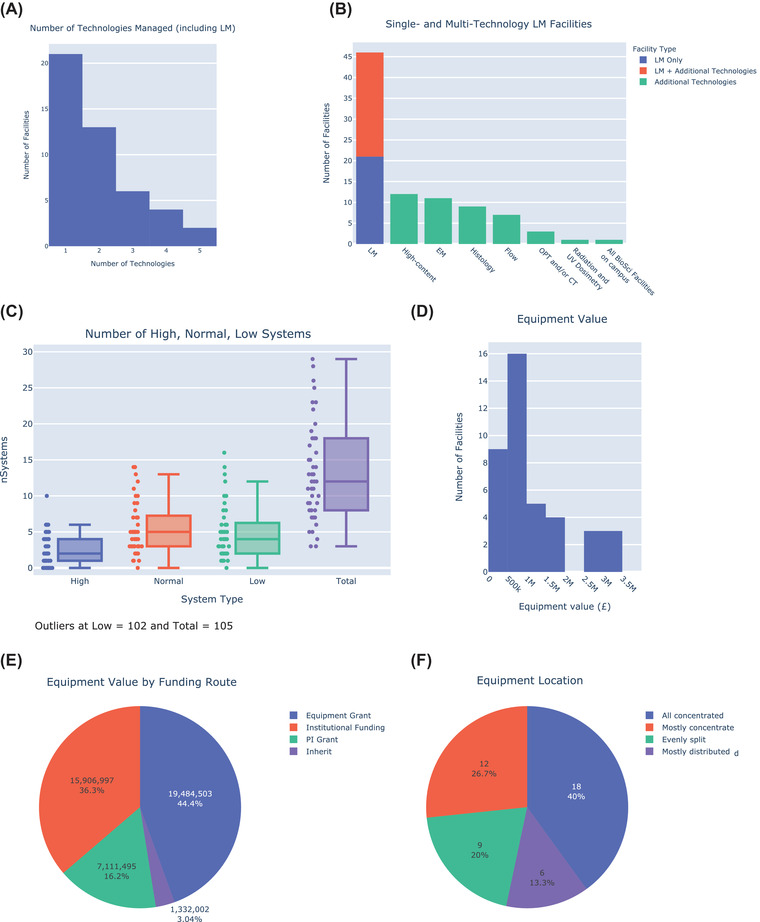
(A) Histogram showing the number of facilities managing multiple technologies. (B) Histogram showing the different technologies managed in addition to LM by UK imaging facilities. (C) Box plot showing the number of high‐end systems (e.g. STED, OMX, Palm etc., FLIM, FCS, 2‐photon with SHG or other specials, light sheet, laser capture microdissection); normal systems (e.g. confocal, TIRF, SD, ratio‐imaging, wide‐field with deconvolution) and low‐end systems (e.g. wide‐field, stereo microscopes, biostation) found in UK LM facilities. Outlying points have been omitted for display clarity. (D) Histogram of equipment value entering UK LM facilities over the past 5 years. (E) Pie chart showing the value of equipment entering UK LM facilities through different routes. (F) Pie chart showing the location of equipment managed by core facilities

More than half of UK LM facilities (25/46) manage more than one major technology, with 13 facilities managing 2 technologies and 12 facilities managing 3 technologies or more (Figure 3A). The most common technologies managed in conjunction with light microscopy are high‐content imaging (*n* = 12), electron microscopy (*n* = 11), histology (*n* = 9) and flow cytometry (*n* = 7) (Figure 3B). UK LM facilities vary widely in the type and number of imaging systems they manage (Figure 3C). Twenty‐five per cent of all facilities have a total of 8 systems or less, 25% have 18 systems or more, with the remaining 50% having between 9 and 17 systems. Facility Heads were asked to categorise their imaging equipment as high‐end, normal and low‐end systems using the following guidelines: high‐end systems, for example STED, OMX, Palm etc., FLIM, FCS, 2‐photon with SHG or other special features, light sheet, laser capture microdissection; normal systems, for example, confocal, TIRF, SD, ratio‐imaging, wide‐field with deconvolution and low‐end systems, for example, wide‐field, stereo microscopes, biostation. The median number of high‐end systems was 2, of normal systems was 5 and of low‐end systems was 4. Over 1/3 of all facilities (17/46) had 0 or 1 high‐end systems. To estimate the value of equipment under management and the importance of different funding sources, we asked Facility Managers how much equipment had come into the facility through different routes over the last 5 years. Forty‐five LM facilities reported having received approximately £44 million in equipment (Figure 3D). Around one‐third of facilities (35%) had received between £0.5–1 million in equipment whereas 13% had received £2.5–3.5 million. The largest single source was equipment grants (44%, Figure 3E) whereas slightly less came from institutional funding (36%). PI grant funding accounted for about 16% of the equipment entering UK LM facilities over the past 5 years. The majority of facility equipment was centrally located, as opposed to being distributed across a building or campus (Figure 3F).

LM facility management typically makes use of easily quantifiable metrics such as hours of instrument booking, number of users and number of training sessions. Such information is readily available from the equipment booking database at the operational heart of most LM facilities and easily accessible to most core facility managers. The GerBi survey of 2016^2^ suggested that a user to staff ratio of approximately 45:1 was common across many German LM facilities. This intriguing metric suggested there might be a characteristic proportion between an LM facility and the local research environment it serves. We were therefore curious to see if there were similar correlations in the United Kingdom among this or possibly other operational metrics, such as the number of staff, users, systems, training sessions or hours of equipment use.

**FIGURE 4:**
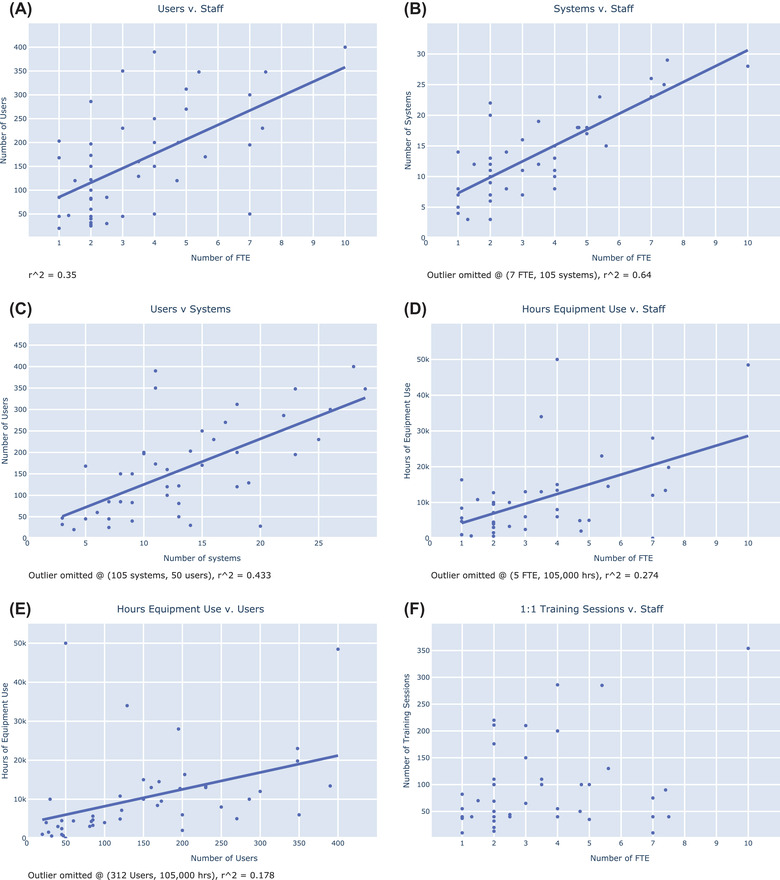
Scatter plots showing the number of (A) facility users and staff, (B) systems and staff, (C) users and systems, (D) hours of equipment use and staff, (E) hours of equipment use and users and (F) 1:1 training sessions and staff across UK LM facilities. Outlying points have been omitted from the analysis as indicated. Trendline parameters are listed in the Supplementary Table S1.

In contrast to the GeBi study, we found weak correlation between the number of users and facility staff (*r* ^2^ = 0.35), with a user to staff ratio closer to 30:1 (Figure 4A and Supplementary Table S1). However, we found relatively strong correlation between the number of systems and staff (*r* ^2^ = 0.64, Figure 4B). The trendline for this graph can be approximated as 2.5 systems per member of staff, plus a *y*‐offset of 5 systems. Weaker correlation was found between the number of users and the number of systems (*r* ^2^ = 0.43, Figure 4C). In general, we found striking spreads among performance metrics related to staff numbers. For example, facilities can be found where two members of staff support from 20 to 280 users (Figure 4A), from 3 to 23 systems (Figure 4B) and from 1000 to 13,000 h of equipment use (Figure 4D). We found poor correlation between the number of users and total hours of equipment use, which is perhaps not surprising given that high‐end, normal and low‐end systems have all been grouped together (Figure 4E). Although the facility reporting the largest number of users also had the largest number of staff (400 users:10 staff), another facility supported nearly as many users with 4 staff. Training numbers also varied strongly, with 4 staff members providing between 40 and 286 one‐to‐one sessions per year in various facilities (Figure 4F). Looking across the performance metrics that were reported, the wide range in the number of users, systems and training sessions supported by 2 staff members is striking. Interpretation of these data, however, is complicated by the differing complexities of system types supported in each facility (from basic wide‐field to super‐resolution).

**FIGURE 5:**
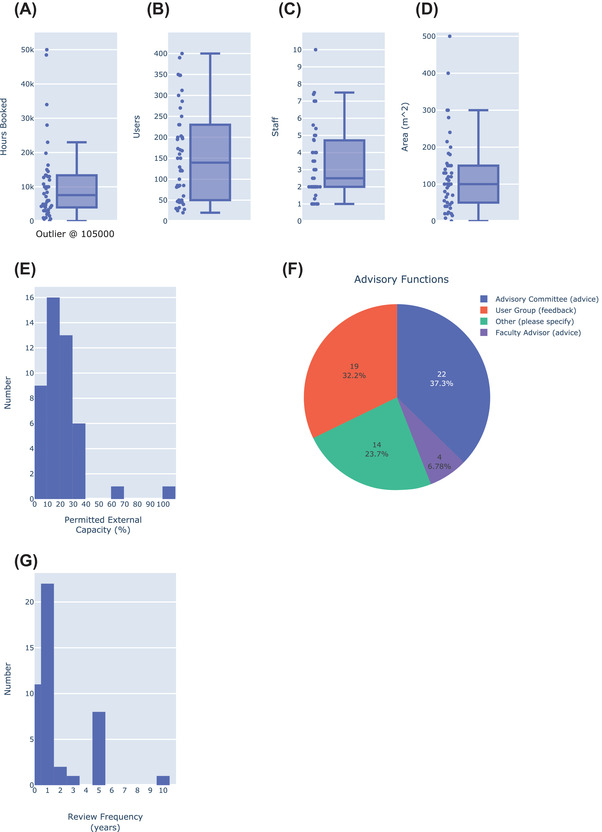
Box plots showing common general metrics of UK LM facility performance including (A) number of hours of equipment booking, (B) number of users, (C) number of staff and (D) facility area. (E) Histogram indicating the fraction of facility capacity available for fee‐paying external users in the context of a program such as Euro‐BioImaging. (F) Pie chart indicating the type of advisory function in place across UK LM facilities. (G) Histogram indicating the frequency of facility review for UK LM facilities

A range of simple metrics, including hours of equipment use, number of users, staff size and facility area, is provided as a baseline for comparison among facility operations (Figure 5A–D). These common metrics indicate a 10‐fold range in the number of hours booked, a 20‐fold range in the number of users supported and a 10‐fold range in the number of staff.

The EuBI model is predicated on the assumption that imaging facilities have some amount of spare capacity which can be made available for guest users from outside the host institution. We tested this assumption by asking UK LM facility managers what percentage of their equipment capacity could be offered to external users. This question examined not only excess capacity, but also the degree to which facilities are permitted to engage with users from outside the host institution. Sixty‐three per cent (29/46) of core facility managers said they could offer 10–29% of their capacity to external users (Figure 5E); 20% (9/46) could offer 9% or less of their capacity to outside users whereas 17% (8/46) could offer 30% or more. Our data thus support the possibility of building national infrastructure on shared capacity, with the caveat that we cannot say whether the excess capacity is available on instruments anyone else wants to use.

Feedback and advice are important inputs for determining the management and direction of an imaging facility. Most UK light microscopy facilities (42 out of 45 responses) have some form of support, monitoring or oversight group, including user groups, advisory committees and faculty advisors (Figure 5F). Advisory committees, which provide direction on operations, were slightly more common than user groups, which provide feedback on operations (37% vs. 32%) and 7 facilities had one of each. Out of 45 facilities only 3 had no formal feedback/advisory input of any kind and 6 had individual feedback mechanisms not found elsewhere. Interestingly, one responder said that the facility review was part of their annual review with their line manager, which seems less than ideal for obtaining meaningful feedback on facility operation (facility reviews should ideally incorporate both user feedback and external ‘fresh eyes’). In addition to receiving input from such groups, most UK LM facilities undergo some form of performance review (Figure 5G). Nearly half are reviewed yearly (22/45), whereas only one‐fifth are reviewed every 5 years (8/45) and one‐quarter (11/46) are not reviewed at all (frequency of review = 0). The performance of 1 facility was reviewed every 10 years, which may be less work for the person being reviewed but seems too long to have a meaningful impact on facility operation in a world of rapidly changing technologies.

## 3 FUNDING

**FIGURE 6:**
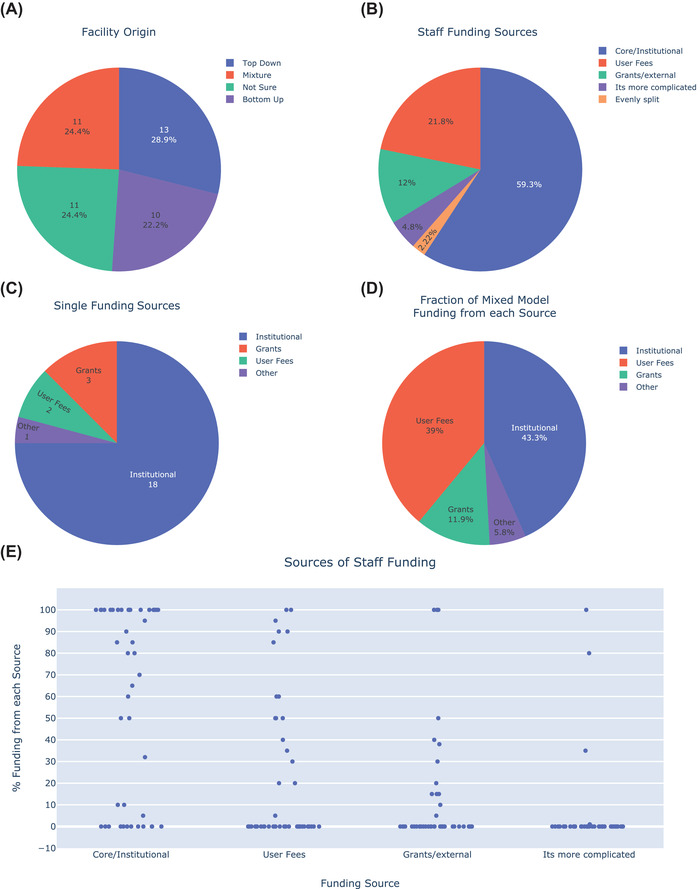
Pie charts indicating (A) the route by which UK LM facilities were formed, (B) the fraction of staff funding coming from different sources for all UK LM facilities, (C) the source of funding for facilities having a single funding source and (D) the fraction of staff funding coming from different sources for those facilities with mixed funding models. (E) Strip plots with the data points underlying B–D

The manner in which facilities are funded influences many aspects of how they are organised and operate. One consideration in facility funding is how the facility was originally established. We hypothesised that a top‐down approach, in which the facility was established through a host institution, could suggest a greater level of institutional commitment to the facility than a bottom‐up approach, in which research groups pool resources to establish the facility. We found that similar numbers of UK LM facilities were established through top down, bottom up and mixed approaches (Figure 6A). We asked Facility Heads to tell us what percentage of their staff salary funding came from typical sources including core funding, user fees and grants. Twenty‐four facilities were funded through a single source whereas 21 facilities relied on funding from multiple sources. Facilities which were formed through a top‐down approach received 78% of their staff funding through core/institutional support and 15% through user fees, whereas bottom‐up facilities received 43% of their staff funding through core/institutional support and 33% through user fees (supporting our hypothesis). Approximately 60% of staff salaries across all UK LM facilities came from core or institutional funding (Figure 6B), and 18 out of 46 facilities said that core / institutional funding was their sole source of staff funding (Figure 6C). Among facilities with multiple funding sources, institutional funding and user fees each provided approximately 40% of staff funding (Figure 6D). The data points underlying staff funding sources are presented in Figure 6E.

**FIGURE 7:**
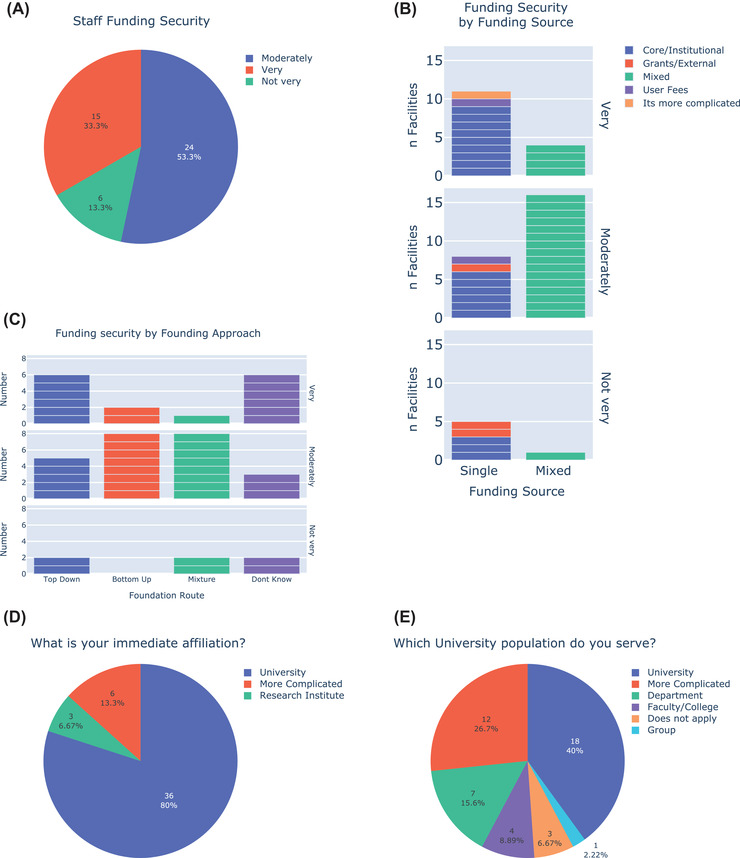
(A) Pie chart indicating the level of staff funding security among UK LM facilities. (B) Faceted bar chart showing the security of staff funding for single‐source and multi‐source facilities, including the funding source for single‐source facilities. (C) Faceted bar chart showing the level of funding security according to the route of facility formation. (D) Pie chart showing the affiliation of UK LM facilities. (E) Pie chart showing the population served by UK LM facilities associated with universities

Funding security is an important aspect of facility management, as well as being important for staff well‐being and career development. We asked UK LM facility managers about the security of their staff funding. Over half (53%) said their staff funding was moderately secure and one‐third (33%) said their funding was very secure (Figure 7A). Core/institutional funding was generally associated with higher security of staff funding. Among facilities that were entirely core/institutionally funded the ratio of very:moderately:not very secure funding was 9:6:3, with half of such facilities reporting very secure funding (Figure 7B). In contrast, the overwhelming majority of facilities funded through multiple sources were moderately securely funded, with a ratio of very:moderately:not very secure funding at 4:15:1. Comparing between single‐ and multi‐funded facilities, approximately 3 times more single‐source facilities were very securely funded (11:4) whereas 2 times more multi‐source facilities were moderately securely funded (16:8, Figure 7B). Among both single and multi‐funding source facilities, higher levels of grant funding were associated with lower funding security. Facilities which entirely relied on grant funding were less secure, and none of the 4 very securely funded mixed source facilities relied on substantial grant funding.

The method of facility foundation was loosely correlated with the level of funding security (Figure 7C). More top‐down founded facilities were very securely funded whereas more bottom‐up facilities were moderately securely funded. This is in line with the observation that top‐down founded facilities had a higher level of core/institutional staff funding, which was generally associated with higher funding security. Overall staff funding security was surprisingly good, and it was reassuring to see that only 13% of UK LM facility managers felt their staff funding was not very secure. It would be interesting to know how these impressions of staff funding security compare to the research community as a whole. Given the role of core facilities in promoting methodological continuity, one might predict that their staff funding is more secure and that this might attract staff into such roles.

80% of UK LM facilities are directly associated with a university, whereas ∼7% are associated with research institutes, and ∼13% have more complicated situations (Figure 7D). Of those facilities associated with universities, 40% serve the university as a whole, ∼10% serve a faculty or college and ∼15% serve a department (Figure 7E). Twenty‐seven per cent describe their user base as being ‘more complicated’, for example involving large numbers of external and/or commercial users.

Approximately 85% of facility managers viewed themselves as having primary or strong influence over the equipment purchased for their facilities and ∼70% viewed themselves as having primary or strong influence over the methods supported by their facilities.

## 4 DISCUSSION

Core funded equipment is a substantial component of the investment made by research funding bodies each year. The LM facilities surveyed here received over £40 million in equipment over the past 5 years. Based on an expected equipment lifetime of 10 years, the purchase value of all equipment under management could easily approach £80 million. Given that our survey represented just over half of the biological LM facilities in the RMS database, the total purchase value of equipment under management across all UK biological LM facilities is likely to be well over £100 million. Most of this equipment has come into the facilities through equipment grants and institutional contribution, rather than PI research projects.

It is important to safeguard and maximise the value of this investment through appropriate core facility management.^10^ Standard metrics of performance can provide useful guidelines for LM facility management and resource allocation.^3^ Such metrics can be helpful for gauging the right level of staff or equipment needed to serve a given user base, and for building a case to obtain resources from local or national funders. They are also important components of facility performance reviews. We therefore carefully examined the relationships among common metrics including the number of users, staff, systems and hours booked. In comparison to the 2016 GerBi survey,^2^ we did not find strong correlation between the number of staff and the number of users they support. Instead, we found the strongest correlation between the number of staff and the number of systems they support. Weaker correlation was found between the number of users and the number of systems.

Our data generally suggest that facilities simply adapt to meet their local demands as well as possible using their existing resources. Striking differences were found in the number of users, systems and hours of equipment use supported by the same number of staff. However, close inspection of the data revealed that similar number of systems (e.g. 10–15) support more users when more staff are present. Our study did not explore the level or quality of support provided by facility staff, which would be expected to vary with the number of users or systems being managed. The strong correlation we observed between staff and system numbers suggests an optimal staffing ratio, which can be used as an argument for under‐staffed facilities to increase staff numbers. Conversely, facilities with user to system ratios well above the trendline could use this information to argue for increased equipment funding from their local institution. A more detailed analysis might also consider the complexity of equipment supported by individual staff members and whether staff, equipment or user ratios correlate with the publication output of the facility or local research environment (see below).

An interesting philosophical point here concerns the nature of dependent and independent variables: does the number of facility users depend on the number of microscopes available, or does the number of microscopes in the facility depend on the number of users needing access? This question cuts to the heart of the search for rules of thumb in facility operation.

We found that 60% of UK LM facilities can offer between 10% and 29% of their capacity to external users, which is a substantial contribution. This suggests that a model for national infrastructure based on shared capacity could be feasible. However, an important caveat is that we cannot say whether the available capacity is on basic equipment to which most people will have local access or advanced systems that would justify travel to use. We did not enquire about available capacity per instrument because this level of granularity was felt to be too burdensome for such a survey. However, it is the sort of information that a facility could be reasonably expected to provide when putting itself forward as part of a national infrastructure proposal. Furthermore, the emphasis on spare equipment capacity misses the equally important question concerning the excess staff capacity needed to support equipment use, which is critical for the use of advanced technologies.

Our data suggest a typical career path where staff enter facility work after a substantial amount of time working in science. Data further support the idea that core facility staff provide scientific expertise in their area and can form an institutional memory for methods employed by different labs over time. Quite a low percentage of staff have come into UK facilities through commercial career paths. This is unfortunate because staff with commercial experience are incredibly valuable in the technical management of equipment (e.g. trouble‐shooting and alignment) and bring important perspectives on the priorities and strategies of industry. To form a more complete picture of career trajectory, it would be important to ask facility managers about the onward destinations of staff who have left the facility. It is encouraging to see that the rate of salary progression for males and females was similar when part‐time workers were excluded. Female salaries appeared more closely clustered around the trendline than male salaries, meaning that there were fewer high‐ and low‐earning outliers among the women compared with the men. The lack of high‐earning female outliers could indicate that women do not fight as hard for higher salaries. Conversely, the lack of low‐earning outliers could indicate that women working in microscopy facilities fight harder against being underpaid. It is unclear from our data whether the lack of women with more than 25 years of experience is due to women having entered LM facility work more recently than men, or whether this is an indication that women have abandoned LM facility work after this time. More detailed analysis of facility career development is needed to clarify these and many other points, for example, the value of part‐time positions and job‐sharing in staff retention. Hopefully we will continue to see higher salaries for senior women in the years to come.

### 4.1 Future studies and survey design

The goal of our survey was to provide an overview of biological microscopy infrastructure in the United Kingdom. We have broadly covered three topics (career development, facility operations and organisation/funding) which could each serve as the basis for a more in‐depth survey. Mindful of the time and effort which goes into filling out such surveys, we tried to ask questions for which the answers would be readily available and avoided topics which would require substantial effort to answer (e.g. number‐crunching, internal research etc.). We hope that our work will help to guide and inform future studies of imaging infrastructure in the United Kingdom and elsewhere. Future surveys will need to balance broad coverage with comprehensive analysis of specific topics, and benefit from experience gained in previous surveys (e.g. try to avoid open response questions, which are difficult to quantify). One approach could be to conduct shorter surveys which comprehensively address single issues.

One of the most important open questions is the relative value‐for‐money of placing imaging equipment in core facilities rather than individual research labs. We found that UK imaging facilities obtained nearly 3 times as much equipment through equipment grants compared to research grants. But how productive is equipment supported through core facilities compared to equipment located in research labs? In the United Kingdom, this question could be approached by examining the outputs assessed by the Research Excellence Framework (REF, www.ref.ac.uk), a national process in which the research outputs of universities are assessed as a means of allocating future research funding. It would be interesting to determine the extent to which different microscopy‐based outputs were achieved through technical resources (staff and/or equipment) located in individual labs or in core facilities. A complimentary approach would be to examine the research outputs derived from similar instruments (e.g. a particular make and model of confocal microscope) placed either in core facilities or research labs. Such information could help to guide the funding and placement of capital equipment, in order to maximise the impact of research funds. As a practical matter, however, any approach to the analysis of staff or equipment productivity will require more accurate recording of the staff and equipment involved in producing research outputs. Facility staff often lament not being acknowledged on publications to which they feel they have made a significant contribution. This is often viewed as a personal gripe; however proper acknowledgement of research contribution is important for two reasons. First, authorship and acknowledgement are critical to staff career progression. Second, lack of acknowledgement on publications also hinders the accurate assessment of how research funds have been used and what outputs they have achieved. The RMS have published guidelines for the acknowledgement of imaging facility contributions to publications,^11^ which we strongly urge the community to adopt. Likewise, it would help to report the specific system (e.g. the serial number) used to acquire data, and ideally its funding source. Improved reporting of facility staff contributions and equipment use will promote more accurate analysis of research funding impact and help guide future resource allocation.

Formulation of a national imaging infrastructure in the United Kingdom requires that we understand the imaging needs of the scientific community and the resources available to meet them. Where do we want to be, where are we now and how do we get from here to there? The goal of this survey is to understand the state of our imaging resources now, which leaves open important questions about scientific needs and how to shape our resources into an appropriate infrastructure to meet them. Setting up infrastructure and keeping it relevant will require a constant dialogue between the scientific community and the funders, to ensure not only that the right methods are selected for support but also that the right level of support is provided through hardware, software, staff, reagents, lab space and access rules.

## Supporting information

Supplementary material

**FIGURE S1**. (A) Bar chart showing the contract type and career track of UK LM facility staff. (B) Word cloud in which the relative frequency of staff job titles is indicated by the size of the font. (C) Scatter plot showing the number of systems and facility area for UK LM facilities.
SUPPLEMENTARY DOCUMENT. The survey questions presented through SurveyMonkey.

TABLE S1. Parameters for the trendlines drawn in Figures 2 and 4A–E, based on the formula Y = mX + b.
