# Supplementary material for: What is the structure of our infrastructure? A review of UK light microscopy facilities
Source: J Microsc. 2022 Jan 10;285(2):55–67. doi: 10.1111/jmi.13076 (PMC9302651; doi:10.1111/jmi.13076)
Supplement: Supplementary file 1 — Supplementary material [file JMI-285-55-s002.pdf]

About you:

1. Is the facility where you work located in the UK?
    - Yes / No
2. What is your job title?
    - Open end
3. What is the job title of your line manager?
    - Open end
4. How many years, including PhD, have you worked in science (to the nearest whole year)?
    - Open end
5. How long have you worked in your current role (to the nearest whole year)?
    - Open end
6. What was your previous role?
    - Commercial (equipment supplier)
    - PhD Student
    - Post-doc
    - Research staff (e.g research assistant/technician/officer)
    - Facility Staff
    - Facility Manager
    - Other (please specify)
7. Do you work:
    - Full-time
    - Part-time
8. What is your current FTE salary in £?
    - Open end
9. For salary normalisation, are you in London or outside?
    - London-based
    - Outside of London
10. Is your job contract:
    - Fixed term
    - Permanent
    - Rolling
11. What career track are you on?
    - Academic
    - Technical
    - Other (please specify)
12. Are you:
    - Female
    - Male
    - Prefer not to say
13. Are you:
    - Academic Head of the Facility
    - Facility Manager
    - Senior staff
    - Junior staff

About your facility:

14. Do you manage:
    - LM
    - EM
    - Flow
    - Histo
    - High-content
    - Other (please specify)
15. How many imaging systems of each type did your facility have in 2019? (Open end)
    - High end systems (eg STED, OMX, Palm etc., FLIM, FCS, 2-Photonwith SHG or other specials, Light Sheet, Laser Capture Microdissection)
    - Normal end systems (eg Confocal, TIRF, SD, Ratio-imaging, WideField with deconvolution, wide-pv convolution)
    - Low end systems (eg Wide-Field, Stereo Microscopes, Biostation)
16. Based on your user-base, do you support:
    - Biological science only
    - Mostly biological science with some material science
    - Equal mix of biological and material science
17. How many hours of equipment use did your facility have in 2019?
    - Open end
18. What percentage of your total capacity could you make available to fee paying outside users (eg in the context of EuroBioImaging)?
    - Open end
19. How many research groups did your facility serve in 2019?
    - Open end
20. How many users did your facility have in 2019?
    - Open end
21. How many one-to-one training sessions did you give in 2019?
    - Open end
22. How many workshops did you give in 2019?
    - Open end
23. How many FTE staff, including image analysts, did your facility have in 2019?
    - Open end
24. How many square-metres does your facility occupy?
    - Open end
25. Is your equipment concentrated within one area of a building or is it distributed across a building or campus?
    - All concentrated in one place
    - Evenly split between concentrated and distributed
    - Mostly concentrated with some distributed
    - Mostly distributed with some concentrated
    - All distributed
26. Does your institution allow you to be Co-Investigator on an external grant application?
    - Yes
    - No

- Don't know

27. How many external grants requesting imaging equipment have you applied for as a named investigator over the past 5 years?
    - 0
    - 1-5
    - 6-10
    - 11-15
    - >15

About your organisation:

28. How many research grant applications have you supported or been associated with over the past 5 years (eg: letter of support, named as manager, costings provided)?
    - 0
    - 1-5
    - 6-10
    - 11-15
    - >15
29. How did your facility get started?
    - Bottom up, eg research groups came together to pool equipment
    - Top down, eg institution set up core facility more or less de novo
    - Mixture of both
    - Don't know, before my time
30. What is your main source of funding for staff salaries? Please breakdown by %, use "0" for none.
    - Core/Institutional Funding
    - User Fees
    - Grants/external funding
    - Evenly split among more than one source
    - Its more complicated than that
31. How secure is your staff funding over the next 5 years?
    - Very
    - Moderately
    - Not very
32. Is your immediate affiliation with a:
    - University
    - Research Institute
    - Its more complicated than that
    - Other (please specify)
33. If you sit within a university which population do you serve?
    - University
    - Faculty/College
    - Department
    - Group
    - Its more complicated than that, eg external users
    - Does not apply
    - Other (please specify)

34. How frequently is the performance of your facility reviewed (in yrs, "0" for never)?
    - Open end
35. Do you have any of the following support/monitoring/oversight groups:
    - User Group (provides feedback on operations)
    - Advisory Committee (provides directions on operations)
    - Faculty Advisor (provides directions on operations)
    - Other (please specify)
36. How much equipment (microscopes, servers etc) in approximate £ has come into your facility through the following routes over the last 5 years: (Open end)
    - Research grants to a PI
    - Equipment grants
    - Institutional funding
    - Inheritance or donation of equipment
37. How much are you able to influence what equipment comes into the facility?
    - Primary decision maker
    - Strong influencer
    - Moderate influence
    - Weak influence
    - No influence
38. How strongly are you able to influence what methods are supported by the facility?
    - Primary decision maker
    - Strong influencer
    - Moderate influence
    - Weak influence
    - No influence
