# Supplementary figures and images for: What is the structure of our infrastructure? A review of UK light microscopy facilities
Source: J Microsc. 2022 Jan 10;285(2):55–67. doi: 10.1111/jmi.13076 (PMC9302651; doi:10.1111/jmi.13076)

## Supplementary Figure

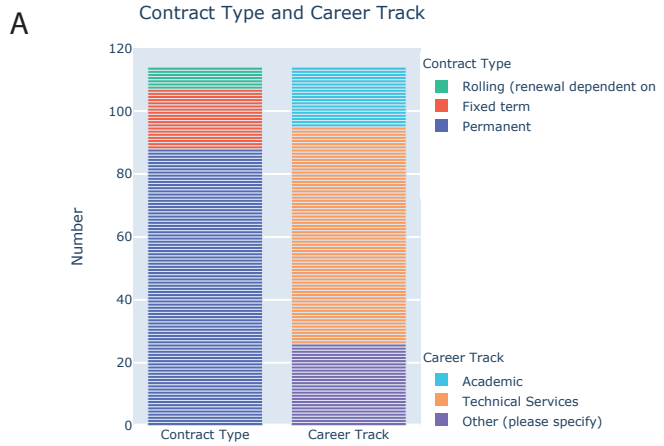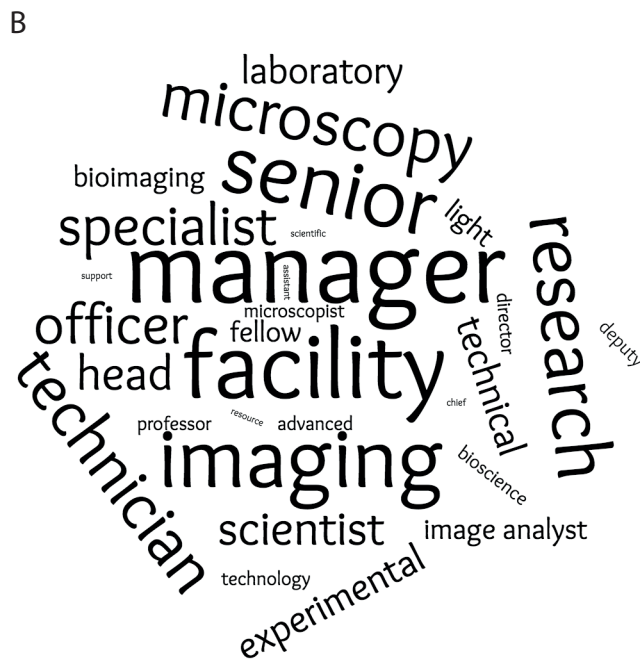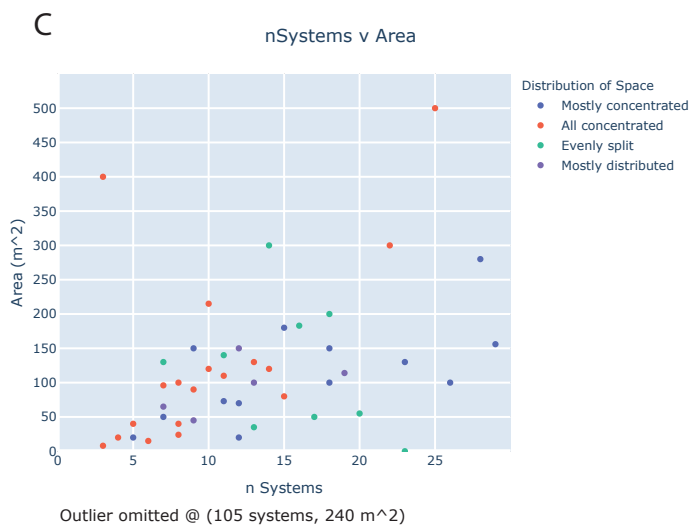

Supplement: Supplementary file 2 — FIGURE S1. (A) Bar chart showing the contract type and career track of UK LM facility staff. (B) Word cloud in which the relative frequency of staff job titles is indicated by the size of the font. (C) Scatter plot showing the number of systems and facility area for UK LM facilities. SUPPLEMENTARY DOCUMENT. The survey questions presented through SurveyMonkey. [file JMI-285-55-s001.pdf]
