# Supplementary material for: What is the structure of our infrastructure? A review of UK light microscopy facilities
Source: J Microsc. 2022 Jan 10;285(2):55–67. doi: 10.1111/jmi.13076 (PMC9302651; doi:10.1111/jmi.13076)
Supplement: Supplementary file 3 — TABLE S1. Parameters for the trendlines drawn in Figures 2 and 4A–E, based on the formula Y = mX + b. [file JMI-285-55-s003.pdf]

Supplemental Table

Fig	Y	m	X	b	r^2
2 - male	Salary (£)	619	Years	30730	0.31
2 - female	Salary (£)	600	Years	30590	0.66
4A	nUsers	30	nFTE	55	0.35
4B	nSystems	2.6	nFTE	4.7	0.64
4C	nUsers	10.6	nSystems	19	0.43
4D	nHours	2710	nFTE	1514	0.274
4E	nHours	43	nUsers	3860	0.178
